# Rare variants in Toll-like receptor 7 results in functional impairment and downregulation of cytokine-mediated signaling in COVID-19 patients

**DOI:** 10.1038/s41435-021-00157-1

**Published:** 2021-12-24

**Authors:** Stefania Mantovani, Sergio Daga, Chiara Fallerini, Margherita Baldassarri, Elisa Benetti, Nicola Picchiotti, Francesca Fava, Anna Gallì, Silvia Zibellini, Mirella Bruttini, Maria Palmieri, Susanna Croci, Sara Amitrano, Diana Alaverdian, Katia Capitani, Simone Furini, Francesca Mari, Ilaria Meloni, GEN-COVID Multicenter Study, Francesca Montagnani, Mario Tumbarello, Ilaria Rancan, Massimiliano Fabbiani, Barbara Rossetti, Laura Bergantini, Miriana D’Alessandro, Paolo Cameli, David Bennett, Federico Anedda, Simona Marcantonio, Sabino Scolletta, Federico Franchi, Maria Antonietta Mazzei, Susanna Guerrini, Edoardo Conticini, Luca Cantarini, Bruno Frediani, Danilo Tacconi, Chiara Spertilli Raffaelli, Marco Feri, Alice Donati, Raffaele Scala, Luca Guidelli, Genni Spargi, Marta Corridi, Cesira Nencioni, Leonardo Croci, Gian Piero Caldarelli, Davide Romani, Paolo Piacentini, Maria Bandini, Elena Desanctis, Silvia Cappelli, Anna Canaccini, Agnese Verzuri, Valentina Anemoli, Manola Pisani, Agostino Ognibene, Alessandro Pancrazzi, Maria Lorubbio, Massimo Vaghi, Antonella D’Arminio Monforte, Federica Gaia Miraglia, Raffaele Bruno, Marco Vecchia, Massimo Girardis, Sophie Venturelli, Stefano Busani, Andrea Cossarizza, Andrea Antinori, Alessandra Vergori, Arianna Emiliozzi, Stefano Rusconi, Matteo Siano, Arianna Gabrieli, Agostino Riva, Daniela Francisci, Elisabetta Schiaroli, Francesco Paciosi, Andrea Tommasi, Pier Giorgio Scotton, Francesca Andretta, Sandro Panese, Stefano Baratti, Renzo Scaggiante, Francesca Gatti, Saverio Giuseppe Parisi, Francesco Castelli, Eugenia Quiros-Roldan, Melania Degli Antoni, Isabella Zanella, Matteo Della Monica, Carmelo Piscopo, Mario Capasso, Roberta Russo, Immacolata Andolfo, Achille Iolascon, Giuseppe Fiorentino, Massimo Carella, Marco Castori, Filippo Aucella, Pamela Raggi, Rita Perna, Matteo Bassetti, Antonio Di Biagio, Maurizio Sanguinetti, Luca Masucci, Alessandra Guarnaccia, Serafina Valente, Oreste De Vivo, Gabriella Doddato, Mirjam Lista, Giada Beligni, Floriana Valentino, Kristina Zguro, Rossella Tita, Annarita Giliberti, Maria Antonietta Mencarelli, Caterina Lo Rizzo, Anna Maria Pinto, Francesca Ariani, Laura Di Sarno, Elena Bargagli, Marco Mandalà, Alessia Giorli, Lorenzo Salerni, Patrizia Zucchi, Pierpaolo Parravicini, Elisabetta Menatti, Tullio Trotta, Ferdinando Giannattasio, Gabriella Coiro, Fabio Lena, Gianluca Lacerenza, Cristina Mussini, Enrico Martinelli, Luisa Tavecchia, Mary Ann Belli, Lia Crotti, Gianfranco Parati, Maurizio Sanarico, Francesco Raimondi, Filippo Biscarini, Alessandra Stella, Tiziana Bachetti, Maria Teresa La Rovere, Serena Ludovisi, Maurizio Bussotti, Simona Dei, Sabrina Ravaglia, Rosangela Artuso, Elena Andreucci, Giulia Gori, Angelica Pagliazzi, Erika Fiorentini, Antonio Perrella, Francesco Bianchi, Paola Bergomi, Emanuele Catena, Riccardo Colombo, Sauro Luchi, Giovanna Morelli, Paola Petrocelli, Sarah Iacopini, Sara Modica, Silvia Baroni, Francesco Vladimiro Segala, Marco Falcone, Giusy Tiseo, Chiara Barbieri, Tommaso Matucci, Davide Grassi, Claudio Ferri, Franco Marinangeli, Francesco Brancati, Antonella Vincenti, Valentina Borgo, Stefania Lombardi, Mirco Lenzi, Massimo Antonio Di Pietro, Francesca Vichi, Benedetta Romanin, Letizia Attala, Cecilia Costa, Andrea Gabbuti, Roberto Menè, Marta Colaneri, Patrizia Casprini, Giuseppe Merla, Gabriella Maria Squeo, Marcello Maffezzoni, Elisa Frullanti, Mario U. Mondelli, Alessandra Renieri

**Affiliations:** 1 Division of Clinical Immunology and Infectious Diseases, Department of Medicine, Fondazione IRCCS Policlinico San Matteo, Pavia, Italy; 2 Medical Genetics, University of Siena, Siena, Italy; 3 Med Biotech Hub and Competence Center, Department of Medical Biotechnologies, University of Siena, Siena, Italy; 4 Department of Mathematics, University of Pavia, Pavia, Italy; 5 University of Siena, DIISM-SAILAB, Siena, Italy; 6 Genetica Medica, Azienda Ospedaliero-Universitaria Senese, Siena, Italy; 7 Department of Hematology Oncology, IRCCS Fondazione Policlinico San Matteo, Pavia, Italy; 8 Core Research Laboratory, ISPRO, Florence, Italy; 9 Department of Internal Medicine and Therapeutics, University of Pavia, Pavia, Italy; 10 Department of Medical Sciences, Infectious and Tropical Diseases Unit, Azienda Ospedaliera Universitaria Senese, Siena, Italy; 11 Unit of Respiratory Diseases and Lung Transplantation, Department of Internal and Specialist Medicine, University of Siena, Siena, Italy; 12 Department of Emergency and Urgency, Medicine, Surgery and Neurosciences, Unit of Intensive Care Medicine, Siena University Hospital, Siena, Italy; 13 Department of Medical, Surgical and Neuro Sciences and Radiological Sciences, Unit of Diagnostic Imaging, University of Siena, Siena, Italy; 14 Rheumatology Unit, Department of Medicine, Surgery and Neurosciences, University of Siena, Policlinico Le Scotte, Siena, Italy; 15 Department of Specialized and Internal Medicine, Infectious Diseases Unit, San Donato Hospital Arezzo, Arezzo, Italy; 16 Department of Emergency, Anesthesia Unit, San Donato Hospital, Arezzo, Italy; 17 Cardio-Thoracic and Neurologic Department, Pneumology Unit and Respiratory Intesive Care Unit, San Donato Hospital, Arezzo, Italy; 18 Department of Emergency, Anesthesia Unit, Misericordia Hospital, Grosseto, Italy; 19 Department of Specialized and Internal Medicine, Infectious Diseases Unit, Misericordia Hospital, Grosseto, Italy; 20 Clinical Chemical Analysis Laboratory, Misericordia Hospital, Grosseto, Italy; 21 Dipartimento di Prevenzione, Azienda USL Toscana Sud Est, Arezzo, Italy; 22 Dipartimento Tecnico-Scientifico Territoriale, Azienda USL Toscana Sud Est, Arezzo, Italy; 23 Clinical Chemical Analysis Laboratory, San Donato Hospital, Arezzo, Italy; 24 Chirurgia Vascolare, Ospedale Maggiore di Crema, Crema, Italy; 25 Department of Health Sciences, Clinic of Infectious Diseases, ASST Santi Paolo e Carlo, University of Milan, Milano, Italy; 26 Division of Infectious Diseases I, Fondazione IRCCS Policlinico San Matteo, Pavia, Italy; 27 Department of Clinical, Surgical, Diagnostic, and Pediatric Sciences, University of Pavia, Pavia, Italy; 28 Department of Anesthesia and Intensive Care, University of Modena and Reggio Emilia, Modena, Italy; 29 Department of Medical and Surgical Sciences for Children and Adults, University of Modena and Reggio Emilia, Modena, Italy; 30 HIV/AIDS Department, National Institute for Infectious Diseases, IRCCS, Lazzaro Spallanzani, Rome, Italy; 31 III Infectious Diseases Unit, ASST-FBF-Sacco, Milan, Italy; 32 Department of Biomedical and Clinical Sciences Luigi Sacco, University of Milan, Milan, Italy; 33 Infectious Diseases Clinic, “Santa Maria” Hospital, University of Perugia, Perugia, Italy; 34 Infectious Diseases Clinic, Department of Medicine 2, Azienda Ospedaliera di Perugia and University of Perugia, Santa Maria Hospital, Perugia, Italy; 35 Department of Infectious Diseases, Treviso Hospital, Local Health Unit 2 Marca Trevigiana, Treviso, Italy; 36 Clinical Infectious Diseases, Mestre Hospital, Venezia, Italy; 37 Infectious Diseases Clinic, ULSS1 Belluno, Italy; 38 Department of Molecular Medicine, University of Padova, Padova, Italy; 39 Department of Infectious and Tropical Diseases, University of Brescia and ASST Spedali Civili Hospital, Brescia, Italy; 40 Department of Molecular and Translational Medicine, University of Brescia, Brescia, Italy; 41 Clinical Chemistry Laboratory, Cytogenetics and Molecular Genetics Section, Diagnostic Department, ASST Spedali Civili di Brescia, Brescia, Italy; 42 Medical Genetics and Laboratory of Medical Genetics Unit, A.O.R.N. “Antonio Cardarelli”, Naples, Italy; 43 Department of Molecular Medicine and Medical Biotechnology, University of Naples Federico II, Naples, Italy; 44 CEINGE Biotecnologie Avanzate, Naples, Italy; 45 IRCCS SDN, Naples, Italy; 46 Unit of Respiratory Physiopathology, AORN dei Colli, Monaldi Hospital, Naples, Italy; 47 Division of Medical Genetics, Fondazione IRCCS Casa Sollievo della Sofferenza Hospital, San Giovanni Rotondo, Italy; 48 Department of Medical Sciences, Fondazione IRCCS Casa Sollievo della Sofferenza Hospital, San Giovanni Rotondo, Italy; 49 Clinical Trial Office, Fondazione IRCCS Casa Sollievo della Sofferenza Hospital, San Giovanni Rotondo, Italy; 50 Department of Health Sciences, University of Genova, Genova, Italy; 51 Infectious Diseases Clinic, Policlinico San Martino Hospital, IRCCS for Cancer Research Genova, Genova, Italy; 52 Microbiology, Fondazione Policlinico Universitario Agostino Gemelli IRCCS, Catholic University of Medicine, Rome, Italy; 53 Department of Laboratory Sciences and Infectious Diseases, Fondazione Policlinico Universitario A. Gemelli IRCCS, Rome, Italy; 54 Department of Cardiovascular Diseases, University of Siena, Siena, Italy; 55 Otolaryngology Unit, University of Siena, Siena, Italy; 56 Department of Internal Medicine, ASST Valtellina e Alto Lario, Sondrio, Italy; 57 Study Coordinator Oncologia Medica e Ufficio Flussi Sondrio, Sondrio, Italy; 58 First Aid Department, Luigi Curto Hospital, Polla, Salerno, Italy; 59 Department of Pharmaceutical Medicine, Misericordia Hospital, Grosseto, Italy; 60 Infectious Diseases Clinics, University of Modena and Reggio Emilia, Modena, Italy; 61 Department of Respiratory Diseases, Azienda Ospedaliera di Cremona, Cremona, Italy; 62 U.O.C. Medicina, ASST Nord Milano, Ospedale Bassini, Cinisello Balsamo (MI), Balsamo, Italy; 63 Istituto Auxologico Italiano, IRCCS, Department of Cardiovascular, Neural and Metabolic Sciences, San Luca Hospital, Milan, Italy; 64 Department of Medicine and Surgery, University of Milano-Bicocca, Milan, Italy; 65 Istituto Auxologico Italiano, IRCCS, Center for Cardiac Arrhythmias of Genetic Origin, Milan, Italy; 66 Istituto Auxologico Italiano, IRCCS, Laboratory of Cardiovascular Genetics, Milan, Italy; 67 Member of the European Reference Network for Rare, Low Prevalence and Complex Diseases of the Heart-ERN GUARD-Heart, Rome, Italy; 68 Independent Data Scientist, Milan, Italy; 69 Scuola Normale Superiore, Pisa, Italy; 70 CNR-Consiglio Nazionale delle Ricerche, Istituto di Biologia e Biotecnologia Agraria (IBBA), Milano, Italy; 71 Direzione Scientifica, Istituti Clinici Scientifici Maugeri IRCCS, Pavia, Italy; 72 Istituti Clinici Scientifici Maugeri IRCCS, Department of Cardiology, Institute of Montescano, Pavia, Italy; 73 Fondazione IRCCS Ca’ Granda Ospedale Maggiore Policlinico, Milan, Italy; 74 Istituti Clinici Scientifici Maugeri IRCCS, Department of Cardiology, Institute of Milan, Milan, Italy; 75 Health Management, Azienda USL Toscana Sudest, Tuscany, Italy; 76 IRCCS C. Mondino Foundation, Pavia, Italy; 77 Medical Genetics Unit, Meyer Children’s University Hospital, Florence, Italy; 78 Department of Medicine, Pneumology Unit, Misericordia Hospital, Grosseto, Italy; 79 Department of Anesthesia and Intensive Care Unit, ASST Fatebenefratelli Sacco, Luigi Sacco Hospital, Polo Universitario, University of Milan, Milano, Italy; 80 Infectious Disease Unit, Hospital of Lucca, Lucca, Italy; 81 Department of Diagnostic and Laboratory Medicine, Institute of Biochemistry and Clinical Biochemistry, Fondazione Policlinico Universitario A. Gemelli IRCCS, Catholic University of the Sacred Heart, Rome, Italy; 82 Clinic of Infectious Diseases, Catholic University of the Sacred Heart, Rome, Italy; 83 Department of Clinical and Experimental Medicine, Infectious Diseases Unit, University of Pisa, Pisa, Italy; 84 Department of Clinical Medicine, Public Health, Life and Environment Sciences, University of L’Aquila, L’Aquila, Italy; 85 Anesthesiology and Intensive Care, University of L’Aquila, L’Aquila, Italy; 86 Medical Genetics Unit, Department of Life, Health and Environmental Sciences, University of L’Aquila, L’Aquila, Italy; 87 Infectious Disease Unit, Hospital of Massa, Massa, Italy; 88 Infectious Diseases Unit, Santa Maria Annunziata Hospital, USL Centro, Florence, Italy; 89 Laboratory of Clinical Pathology and Immunoallergy, Florence-Prato, Italy; 90 Laboratory of Regulatory and Functional Genomics, Fondazione IRCCS Casa Sollievo della Sofferenza, San Giovanni Rotondo (Foggia), Foggia, Italy; 91 University of Pavia, Pavia, Italy

**Keywords:** Immunology, Genetics

## Abstract

Toll-like receptors (TLR) are crucial components in the initiation of innate immune responses to a variety of pathogens, triggering the production of pro-inflammatory cytokines and type I and II interferons, which are responsible for innate antiviral responses. Among the different TLRs, TLR7 recognizes several single-stranded RNA viruses including SARS-CoV-2. We and others identified rare loss-of-function variants in X-chromosomal *TLR7* in young men with severe COVID-19 and with no prior history of major chronic diseases, that were associated with impaired TLR7 signaling as well as type I and II IFN responses. Here, we performed RNA sequencing to investigate transcriptome variations following imiquimod stimulation of peripheral blood mononuclear cells isolated from patients carrying previously identified hypomorphic, hypofunctional, and loss-of-function *TLR7* variants. Our investigation revealed a profound impairment of the TLR7 pathway in patients carrying loss-of-function variants. Of note, a failure in IFNγ upregulation following stimulation was also observed in cells harboring the hypofunctional and hypomorphic variants. We also identified new *TLR7* variants in severely affected male patients for which a functional characterization of the TLR7 pathway was performed demonstrating a decrease in mRNA levels in the *IFNα*, *IFNγ*, *RSAD2*, *ACOD1*, *IFIT2*, and *CXCL10* genes.

## Introduction

Coronavirus disease 2019 (COVID-19), caused by the severe acute respiratory syndrome coronavirus 2 (SARS-CoV-2) [1], has rapidly developed into a global pandemic of enormous consequences. COVID-19 is characterized by a broad spectrum of clinical manifestations in humans, ranging from asymptomatic to mild symptomatic to severe pneumonia accompanied by multiorgan failure [2]. Older age, male sex, hypertension, diabetes, and obesity are all indicators identified as risk factors predisposing to severe disease [2]. In addition, and perhaps underlying some of these indicators, specific genetic factors may more precisely explain the predisposition of some individuals to develop severe disease requiring hospitalization and even admission to intensive care units [2]. Increasing evidence suggests that defects in responsiveness to type I interferons (IFN-I) are of prime importance. Indeed, genetic variants that decrease IFN-I production and the development of anti-IFN-I autoantibodies have been associated with more severe COVID-19 [3–7]. Recently, two studies in young men with severe COVID-19 and no history of major chronic diseases identified rare loss-of-function (LOF) variants in X-chromosomal *TLR7* that were associated with impaired *TLR7* signaling as well as type I and II IFN responses [4, 5]. Another study revealed that at least 3.5% of patients with life-threatening COVID-19 pneumonia had genetic mutations at candidate loci known to be involved in TLR3- and IRF7-dependent induction and amplification of IFN-I [7].

Interferons are rapidly produced following viral infection and induce potent first-line defense mechanisms against viruses that are key in host–virus standoff [8]. The initial sensing of pathogens is mediated by innate pattern recognition receptors that include Toll-like receptors (TLRs). The intracellular signaling cascades triggered by TLRs lead to the transcriptional expression of inflammatory mediators that coordinate the elimination of pathogens and infected cells. Interestingly, among the different TLRs, TLR7 binds to single-stranded RNA viruses, such as influenza A virus, HIV-1, hepatitis C virus, HBV RNA intermediates, and SARS-CoV-2 as well as binding to synthetic guanine-rich RNA sequence analogs such as imiquimod (IMQ) [9–13]. Upon virus infection or agonist stimulation, TLR7 dimerizes in the endosome to initiate TLR7-mediated MyD88 signal transduction, resulting in the activation of mitogen-activated protein kinase cascades and NF-κB [14]. Signaling in human immune cells by TLR7 has been documented to trigger production of pro-inflammatory cytokines, including tumor necrosis factor α (TNF-α), interleukin (IL)-6, IL-1β, and IL-12 as well as IFN-I. IFN-I regulates a range of immune responses through the IFN-I receptor, resulting in the transcription of hundreds of IFN-stimulated genes (ISGs) whose joint action leads to the generation of an “antiviral state” [8, 14].

To gain insight into TLR7-linked mechanisms of severe COVID-19, we performed RNA sequencing (RNA-Seq) to carefully characterize transcriptome variations following IMQ stimulation of peripheral blood mononuclear cells (PBMC) isolated from patients carrying previously identified LOF *TLR7* variants [5]. In addition, we found new *TLR7* variants in severely affected males for which functional characterization of the pathway was also performed.

## Results and discussion

**Fig. 1:**
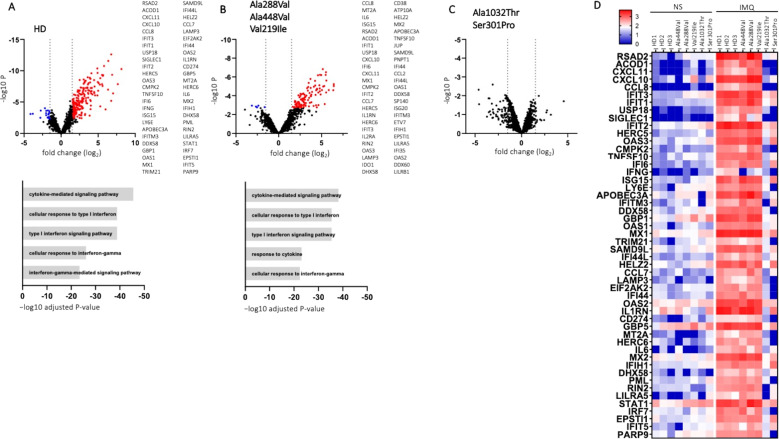
DEGs in PBMC from HDs and patients carrying *TLR7* variants stimulated with IMQ. **A**–**C** Volcano plots showing DEGs. Red dots show upregulated genes (log2FC ≥ 1.5 with adjusted *p* value ≤ 0.05) and blue dots represent downregulated genes (log2FC ≤ −1,5; adjusted *p* value ≤ 0.05). The DEGs with the top 50 absolute FC are reported. **A** Three healthy donors. **B** Patients (*n* = 3) carrying the Ala288Val, Ala488Val, and Val219 Ile variants. **C** Patients carrying Ala1032Thr (*n* = 1) and Ser301Pro (*n* = 1) variants. Gene Ontology biological process terms significantly overrepresented among the genes increased by IMQ are shown in the lower panel in (**A**, **B**, **D**). Heatmap of logCPM values for the top DEGs in HDs and patients carrying *TLR7* variants after IMQ stimulation. IMQ imiquimod, NS non stimulated.

To study more deeply the functional effects of *TLR7* variants, after TLR7 stimulation with IMQ in comparison with unstimulated cells, we performed RNA-Seq experiments on PBMC from healthy donors (HDs) and from patients carrying the functionally hypomorphic variants Ala288Val and Ala448Val, the hypofunctional variant Val219Ile, and the LOF variants Ala1032Thr and Ser301Pro. As shown in Fig. 1A, B, we observed several differentially expressed genes (DEGs) in the HDs as well as in the patients carrying hypomorphic and hypofunctional variants. In contrast, when LOF variants were analyzed, no DEGs were found (Fig. 1C). Specifically, TLR7 stimulation induced a strong response in HDs with 211 genes significantly upregulated (log2 fold change (FC) ≥ 1.5; adjusted *p* value ≤ 0.05) and 19 downregulated genes (log2FC ≤ −1.5; adjusted *p* value ≤ 0.05) compared with unstimulated PBMC. The genes displaying the top 50 absolute FC are listed in Fig. 1A. We used the Gene Ontology (GO) database to perform GO-biological process enrichment analysis of DEGs. Cytokine-mediated signaling and cellular response to interferons were upregulated pathways in HDs (Fig. 1A, lower panel). Patients carrying hypomorphic or hypofunctional variants displayed 108 upregulated genes (log2FC ≥ 1.5; adjusted *p* value ≤ 0.05) and 5 downregulated genes (log2FC ≤ −1.5; adjusted *p* value ≤ 0.05), most of which were the same observed in HDs (Fig. 1B). The GO-biological process enrichment analysis identified the same upregulated pathways (Fig. 1B, lower panel). Interestingly, RNA-seq analysis in patients carrying LOF variants showed that none had genes with an adjusted *p* value ≤ 0.05 (Fig. 1C), suggesting a profound impairment of the *TLR7* pathway. As shown in the heat map (Fig. 1D), for most of the 50 genes with the highest FC in HDs after IMQ stimulation we noticed a significant upregulation in patients carrying hypomorphic and hypofunctional variants but not in patients with LOF variants. A notable exception was *IFNγ* for which a failure to induce upregulation following stimulation was also observed in cells harboring the hypofunctional and hypomorphic variants. It has been shown that at around day 10 in subjects with COVID-19, IFN-I decreased while IFNγ remained stable [15], promoting the resolution of lung inflammation. Therefore, administration of IFN-I might be considered a therapeutic option for TLR7 mutated patients. The efficacy of IFN-I therapy would depend on whether it is administered early in the course of the disease. Patients with a severe course of COVID-19 are usually admitted to the hospital after a few days at home making it difficult to identify those in need of IFN-I treatment. Indeed, inappropriate administration of IFN-I to the wrong patients or at the wrong time point could be counterproductive by triggering the cytokine storm. A more attractive therapeutic option would be IFNγ, which is not only useful in patients with hypomorphic mutations but, in addition, can stabilize the inflammatory response and does not require timely administration.

As shown in Fig. 1, in HDs and in patients carrying hypomorphic and hypofunctional variants, but not in patients carrying LOF variants, TLR7 engagement triggered an antiviral response upregulating typical antiviral ISGs (*RSAD2*, *APOBEC3A*, *HERC5*, *OASs*, *MXs*, *IFITs*, and *IFITMs* family genes) as well as proinflammatory cytokine and chemokine genes (*IL6*, *CXCL10*, *CXCL11*, *CCL8*, *CCL2*, *CCL7*) [8]. Interestingly, IL6 and CXCL10 were found to be involved in the mechanisms sustaining the cytokine-storm, a peculiar aspect of SARS-COV-2 infection which, at least in severe cases, is responsible for diffuse alveolar damage and multi-organ failure [16]. Furthermore, IFITMs inhibit cellular entry of SARS-CoV and MERS-CoV [17]. We also observed upregulation of *CCL2* and *CCL7*, two pivotal chemokines for monocyte recruitment, both of which were found enriched in bronchoalveolar fluid (BALF) from patients with severe COVID-19 [18, 19]. Moreover, the *TNFSF10* gene (*TRAIL*), an apoptosis-related gene that was previously found to be upregulated in BALF and PBMC from COVID-19 patients [20], was also upregulated after IMQ stimulation in HD and in patients carrying hypomorphic and hypofunctional variants. Meanwhile, we observed a marked induction of negative regulators (such as *USP18*, *IL1RN*, and *ACOD1*), suggesting stimulation of negative feedback loops. The functional status of the cells was evaluated by stimulating PBMC from patients and HDs with the TLR4 agonist lipopolysaccharide (LPS). Intracellular production of IL6 was evaluated in monocytes (as shown in Supplementary Fig. 1). The frequencies of CD3^−^CD14^+^IL6^+^ cells were comparable in patients and HDs, indicating that cells from patients harboring *TLR7* variants were functionally active. IFNα and IFNγ protein production was evaluated in the supernatant of PBMC from HDs and from a small number of patients after TLR7 engagement. The data showed a trend toward a lower production of IFNα as well as IFNγ proteins in patients carrying LOF TLR7 variants. The patient carrying the hypomorphic variant Ala288Val showed a reduced, though not statistically significant, production of IFNα protein after TLR7 engagement (Supplementary Fig. 2). Overall, the transcriptomic profile of cells harboring LOF *TLR7* variants showed a wide deficiency of ISGs while both hypomorphic and LOF mutations displayed a reduction of IFNγ transcription.

**Fig. 2:**
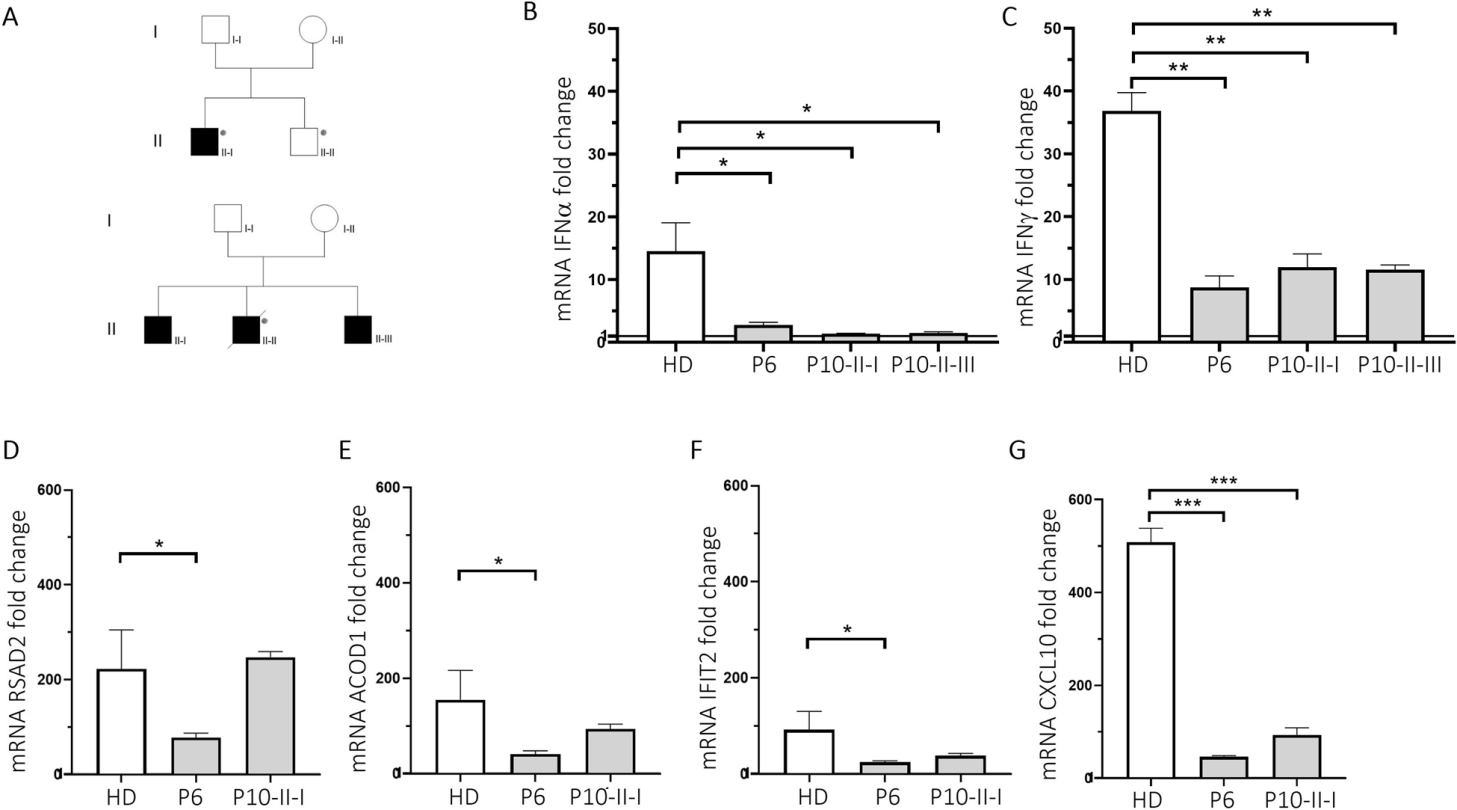
Gene expression analysis in PBMC of patients carrying *TLR7* variants stimulated with IMQ. **A** Pedigree of P6 (**A** upper panel) and P10 (**A** lower panel) shows the segregation of the variant within the family. Squares represent male family members; circles, females. Black symbols indicate individuals harboring the *TLR7* variant. Individuals infected by SARS-CoV-2 are indicated by a virus symbol () close to the individual symbol. **B**–**G** PBMC from COVID-19 patients and four unaffected male controls (HDs) were stimulated with IMQ at 5 μg/mL or cell culture medium. A quantitative PCR assay was performed and 2^−ΔΔCt^ was calculated using *HPRT1* as the housekeeping gene. Fold change in mRNA expression of genes induced by IMQ with respect to cell culture medium was calculated: *p < 0.05; **p < 0.01; ***p < 0.001.

We next extended the analysis to two additional rare *TLR7* variants: the already reported Arg920Lys variant (P6) [5], and the new Asp41Glu (P10) variant predicted to be deleterious from in silico analysis (Fig. 2A). The two variants were identified in two severely affected male patients aged 49 and 79 years, one in each. In order to functionally characterize the TLR7 pathway, we performed a gene expression profile analysis of PBMC from patient P6 and from two relatives of patient P10 following stimulation with IMQ. We found a statistically significant decrease in mRNA levels for *IFNα* and *IFNγ* genes in P6, P10-II-I, and P10-II-III compared with HDs (Fig. 2B, C). We further analyzed some of the genes showing the highest FC in the HDs transcriptomic profile and observed a significant decrease of mRNAs encoding for *RSAD2*, *ACOD1*, and *IFIT2* genes in P6, demonstrating a profound impairment of the TLR7 signaling pathway in response to TLR7 stimulation (Fig. 2D–F). Of note, it was reported that RSAD2, in addition to the role of direct suppressor of viral replication, promotes TLR7- and TLR9-mediated production of IFNα. [21]. Moreover, we observed that CXCL10 mRNA was markedly reduced in P6, P10-II-I, and P10-II-III compared with HDs (Fig. 2G). Overall, our data expand previous findings on the TLR7 role in rare Mendelian forms of COVID-19 and provide further insights into the altered pathways that might contribute to disease severity.

## Materials and methods

Male COVID-19 patients were selected from the Italian GEN-COVID cohort [5]. Cases were selected according to the following inclusion criteria: (i) male gender; (ii) young age (<60 years); and (iii) detection of rare variants in *TLR7*. Exclusion criteria were: (i) SARS-CoV-2 infection not confirmed by PCR. Relatives of patients 6 and 10 were contacted to obtain a blood sample. Segregation analysis of the variants was performed with Sanger sequencing on an ABI3130 Genetic Analyzer. PBMC isolation, IMQ stimulation, and qPCR were performed as previously described [5]. The primers used are listed in Supplementary Table 1. Supernatants of PBMCs stimulated with IMQ, LPS, or medium alone were measured for IFNα (Invitrogen) and IFNγ production (Bio-Techne) according to the manufacturer’s instructions. PBMC were stimulated in vitro with LPS at 1 µg/ml for 4 h, then IL6 production was examined in CD3^−^ CD14^+^ cells by flow cytometry. Briefly, 3 × 10^5^ PBMC were stained with anti-CD3 BV605 and anti-CD14 BB700 mAbs, fixed and permeabilized with the BD Cytofix/Cytoperm kit in the presence of anti-IL6 BV421 (Becton Dickinson) according to the manufacturer’s instructions. RNA quality was assessed by Fragment Bioanalyzer (Agilent); all samples exhibited RNA quality numbers greater than 8. The libraries for RNA-seq were performed according to the Illumina TruSeq Stranded mRNA Library preparation protocol and sequenced in multiplex with HiSeq 2500 platform (Illumina) in 50 nucleotides, paired-end read configuration. Sequencing data were analyzed using the BioJupies platform [22].

WES and Genotype (GWAS) data were generated within the GEN-COVID Data Repository (GCGDR). In order to be able to store and analyze the massive amount of genomic data generated with the analysis of the entire cohort of samples populating the biobank, we relied on the NIG. External users can upload and analyze data using the NIG pipeline by registering and creating a specific project. A section dedicated to COVID-19 samples has been created within the NIG database (http://nigdb.cineca.it/) that provides variant frequencies as a free tool for both clinicians and researchers.

## Supplementary information

Supplementary Information

Supplementary Figure 1

Supplementary Figure 2

Supplementary Table 1
