# Supplementary material for: Rare variants in Toll-like receptor 7 results in functional impairment and downregulation of cytokine-mediated signaling in COVID-19 patients
Source: Genes Immun. 2021 Dec 24;23(1):51–6. doi: 10.1038/s41435-021-00157-1 (PMC8703210; doi:10.1038/s41435-021-00157-1)
Supplement: Supplementary file 1 — Supplementary Information [file 41435_2021_157_MOESM1_ESM.docx]

**Supplementary figure 1. Intracellular production of IL6 in monocytes after LPS stimulation**

PBMC from patients and HDs were stimulated with the TLR4 agonist lipopolysaccharide (LPS) for 4 hours (left panel) or with medium alone (NS). The intracellular production of IL6 was evaluated in CD3^-^ CD14^+^cells by flow cytometry, as shown in the representative dot plots (right panel).

**Supplementary figure 2.** IFN𝛼 and IFNγ production after IMQ stimulation. PBMC from HDs and patients were stimulated with IMQ at 5 μg/mL, LPS or cell culture medium (NS). The production of IFN𝛼 (left panel) and IFNγ protein (right panel) was evaluated in supernatant by ELISA.
