# Supplementary figures and images for: Rare variants in Toll-like receptor 7 results in functional impairment and downregulation of cytokine-mediated signaling in COVID-19 patients
Source: Genes Immun. 2021 Dec 24;23(1):51–6. doi: 10.1038/s41435-021-00157-1 (PMC8703210; doi:10.1038/s41435-021-00157-1)

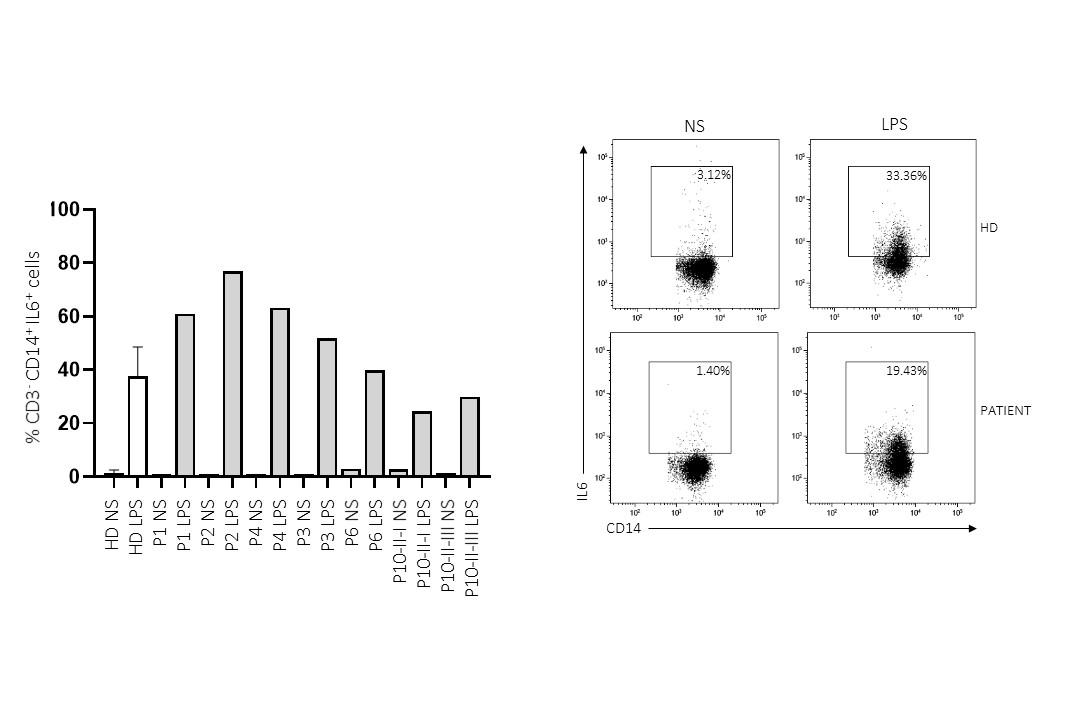

Supplement: Supplementary file 2 — Supplementary Figure 1 [file 41435_2021_157_MOESM2_ESM.jpg]

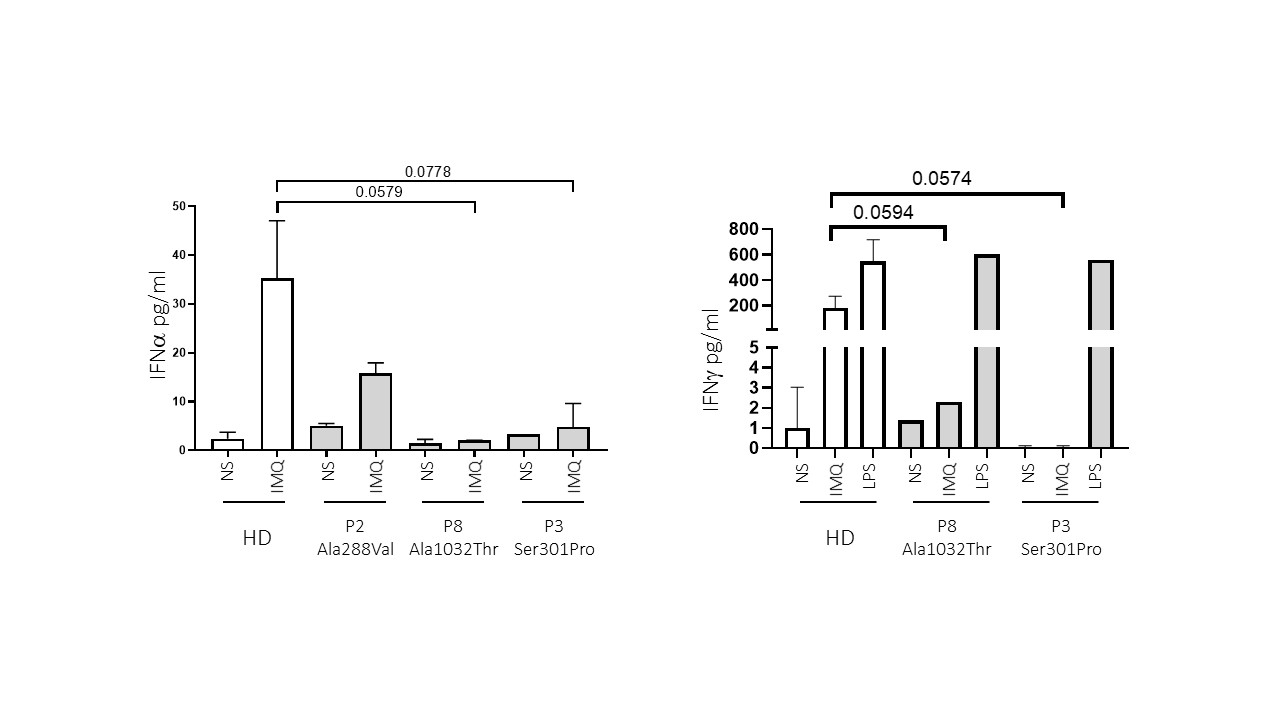

Supplement: Supplementary file 3 — Supplementary Figure 2 [file 41435_2021_157_MOESM3_ESM.jpg]
